# Risk, protective, and biomarkers of dementia in Indigenous peoples: A systematic review

**DOI:** 10.1002/alz.13458

**Published:** 2023-09-25

**Authors:** Huong X. T. Nguyen, Kate Bradley, Bridgette J. McNamara, Rosie Watson, Roslyn Malay, Dina LoGiudice

**Affiliations:** ^1^ Department of Medicine Royal Melbourne Hospital Melbourne Victoria Australia; ^2^ Department of Population Health and Immunity Walter and Eliza Hall Institute of Medical Research Melbourne Victoria Australia; ^3^ Centre for Epidemiology and Biostatistics University of Melbourne Victoria Australia; ^4^ Barwon South‐West Public Health Unit Barwon Health Geelong Victoria Australia; ^5^ Western Australian Centre for Health and Ageing University of Western Australia Perth WA Australia

**Keywords:** biomarkers, dementia, first nations, Indigenous, prevention

## Abstract

**INTRODUCTION:**

Dementia is an emergent health priority for Indigenous peoples worldwide, yet little is known about disease drivers and protective factors.

**METHODS:**

Database searches were conducted in March 2022 to identify original publications on risk, protective, genetic, neuroradiological, and biological factors related to dementia and cognitive impairment involving Indigenous peoples.

**RESULTS:**

Modifiable risk factors featured across multiple studies include childhood adversity, hearing loss, low education attainment, unskilled work history, stroke, head injury, epilepsy, diabetes, hypertension, hyperlipidemia, depression, low BMI, poor mobility, and continence issues. Non‐modifiable risk factors included increasing age, sex, and genetic polymorphisms. Education, ex‐smoking, physical and social activity, and engagement with cultural or religious practices were highlighted as potential protective factors. There is a paucity of research on dementia biomarkers involving Indigenous peoples.

**DISCUSSION:**

Greater understanding of modifiable factors and biomarkers of dementia can assist in strength‐based models to promote healthy ageing and cognition for Indigenous peoples.

## 1 INTRODUCTION

Dementia is characterized by a progressive decline in cognition that translates into the loss of functional abilities and independence. It is a growing global health challenge with impacts on individuals, families, and systems of care. There are currently more than 50 million people living with dementia worldwide, with numbers estimated to exceed 152 million by 2050.^1^ For Indigenous peoples, who comprise 6% of the world's population, dementia is an emerging health priority in light of increasing survivorship to old age.^2^

The United Nations recognizes Indigenous peoples as those who, by self‐identification, have a “historical continuity with a given region prior to colonization and strong link to their lands” and maintain “distinct languages, cultures, beliefs, and knowledge systems” from the mainstream or dominant society.^3^ Worldwide, Indigenous peoples often experience inequities in social and biological determinants of health as a legacy of colonization and corrosion of traditional ways of life. Adverse social determinants of disease such as intergeneration trauma, racial discrimination, cultural disconnection, land dispossession, and reduced access to health services and education lead to a greater burden of non‐communicable chronic diseases, including dementia.^2^

There is currently no widely available and clinically effective disease‐modifying treatment for dementia and as such, modification of risk and protective factors are crucially important in strategies to promote healthy aging and prevent cognitive decline. The 2020 Lancet Commission report on dementia, prevention, intervention, and care suggests that, at a population level, up to 40% of dementia could be preventable by eliminating 12 risk factors.^4^ It identified less education in early life, hypertension, hearing impairment, smoking, obesity, depression, physical inactivity, diabetes, social isolation, traumatic brain injury, excessive alcohol consumption, and air pollution as key risk factors in the development of cognitive problems.^4^ These risk factors feature in the health and social profile of Indigenous peoples however, a greater understanding of the risk and protective factors unique to specific Indigenous groups and those shared across First Nation populations is important in developing more effective approaches to decrease the burden and sequalae of dementia.^2^

The pathogenesis of dementia often begins decades before the onset of symptoms and changes in brain pathology detectable in midlife may offer insights into dementia risk in the future. Gray and white matter changes on magnetic resonance imaging (MRI) can support clinical diagnosis and assist in the differentiation of one type of dementia from another. Alzheimer's disease‐specific biomarkers such as amyloid beta and tau have been demonstrated using positron emission tomography (PET) imaging and in fluids (cerebrospinal fluid and plasma).^5^, ^6^ These disease‐specific biomarkers, alongside microvascular and inflammatory biomarkers involved in cellular signaling, immune responses, neuronal support, and apoptosis have also been studied in peripheral blood.^7^ Advances in scientific understanding of genetic, neuroimaging, and biological markers augment understanding of dementia phenotype as a complex interaction between genetics, biology, and exposures across the life‐course. They may, in the future, have greater utility in clinical practice to identify those at higher risk of a neurodegenerative process, improve timely and accurate diagnosis, and monitor disease progression and effects of interventions.^8^ Despite great strides in the field, the relationship between biomarkers and ethnoracial factors remains understudied. Large studies on cognition and aging such as the Australian Imaging Biomarkers and Lifestyle Study of Ageing (AIBL) and Alzheimer's disease neuroimaging initiative (ADNI) from North America, do not contain a broad representation of ethnoracial populations.^9^, ^10^

Our aim was to describe and evaluate the evidence base for risk, protective, and biomarkers associated with dementia and cognitive impairment in Indigenous peoples around the world. We also provide an update of prevalence and incidence of dementia and cognitive impairment in Indigenous populations. We build on existing systematic reviews on this subject in our synthesis of emerging research.^11^, ^12^, ^13^ To our knowledge, this will be the first review of risk, protective *and* biomarkers of dementia and cognitive impairment involving Indigenous peoples.

**RESEARCH IN CONTEXT**

1. **Systematic review**: The authors reviewed the evidence base for risk, protective and biomarkers of dementia in Indigenous peoples worldwide. This is the first review of biomarkers of dementia and cognitive impairment involving Indigenous peoples.
2. **Interpretation**: Database searches yielded 1567 titles for screening, 132 articles for review and 39 were included in the systematic review. Prevalence rates of dementia varied widely from 0.6% to 37.2% with rates of MCI/CIND between 4.4% and 27.4% for those aged ≥60 years. There are many shared determinants of health and illness between Indigenous populations however, appreciation of unique population profiles can assist in the development of targeted strength‐based models to promote cognitive health and prevent impairment over the life‐course.
3. **Future directions**: Increased research is needed on modifiable protective factors and biomarkers of dementia involving Indigenous peoples.

## 2 METHODS

### 2.1 Search strategy

The protocol for this review was registered in PROSPERO (CRD42020207449). A literature search of Medline, Embase, CINAHL, Global Health, Emcare, and PsycINFO databases was conducted using the OVID platform on the 15th of March 2022. Search terms included all descriptors related to: *Indigenous persons*, *dementia*, *cognitive impairment*, *prevalence*, *incidence*, *risk factor*, *protective factors*, *genetic markers*, *neuroimaging*, and *biomarkers*. Indigenous population search terms were based on the Lancet‐Lowitja Institute Global Collaboration publication on the health of Indigenous and tribal peoples and checked against works in the same canon.^2^ This review was limited to age‐related dementias such as Alzheimer's dementia (AD), vascular dementia (VaD), Lewy body dementia (LBD), and fronto‐temporal dementia (FTD), and does not address dementia secondary to primary neurological conditions (e.g., amyotrophic lateral sclerosis), retroviruses (e.g., human immunodeficiency virus), or other pathogens (e.g., syphilis). Cognitive impairment encompassed cognitive impairment not dementia (CIND) and mild cognitive impairment (MCI) diagnoses and pertained to those with evidence of cognitive decline from baseline without functional loss to meet a diagnosis of dementia. CIND and MCI were included in this review as they may represent an early stage of dementia with shared risk or protective factors. The full search strategy is detailed in Appendix SA. The review is reported according to the Preferred Reporting Items for Systematic Reviews and Meta‐Analyses (PRISMA).^14^

### 2.2 Study selection and data extraction strategy

References from the literature search underwent screening by two reviewers (H.N. and K.B.) based on predefined inclusion and exclusion criteria. We included original studies that focused on a recognized Indigenous population that comprised >10% of the study cohort. Indigenous peoples were chosen with reference to the UN statement and the Lancet‐Lowitja Institute Global Collaboration publication on the health of Indigenous and tribal peoples across all world continents, except Antarctica, with emphasis on self‐identification, connection to country, and acceptance within a cultural community.^2^, ^3^ Studies included dementia and/or cognitive impairment as outcome measures of interest and contained information on: prevalence, incidence, risk factors, protective factors, and/or biomarkers. Single case studies, dissertation abstracts, reviews and articles not available in English through publication or in translation were excluded. Risk factors are defined as identifiable events or conditions associated with an increased probability of disease. Protective factors are those associated with a decreased probability of disease. Studies involving genetic markers such as apolipoprotein E (*APOE*) and biological markers such as homocysteine were included when they were studied in relation to the outcomes of interest. Biomarkers were taken to be any molecule in the brain or biological fluids associated with a disease state that facilitates its diagnosis. Structural and functional neuroimaging markers that are important in diagnosis as a marker of disease states of interest were also included. These encompass descriptors of regional cerebral atrophy and white matter changes on MRI as well as fluorodeoxyglucose and amyloid radiotracer avidity using functional and molecular imaging modalities. Discrepancies were resolved through discussion with a third reviewer (D.L.) to achieve consensus.

### 2.3 Quality assessment

Systematic appraisal of included articles was performed using a checklist adapted from the National Institute of Health (NIH) Study Quality Assessment Tool for Observational Cohort and Cross‐Sectional Studies.^15^ Engagement, governance, or co‐authorship with Indigenous people/communities/organizations and reporting of positive outcomes (strength‐based model) was included to assess the reporting of Indigenous health research in line with the CONSIDER Statement and Aboriginal and Torres Strait Islander quality appraisal tool.^16^, ^17^ Studies were assigned a quality rating of good, fair, or poor (Appendix SB). Discrepancies were resolved through discussion with the third reviewer (D.L.).

### 2.4 Data synthesis

The articles were observed to be highly heterogeneous and focused on different aspects of the research question. As such, pooling of the results in a meta‐analysis was not possible due to substantial variance in focus, methods, and outcome measures employed. The findings were therefore synthesized in descriptive form. Prevalence rates were not standardized for comparison between Indigenous populations due to the lack of an appropriate reference cohort. Indeed, using any one Indigenous group as a reference would assume the sameness of all Indigenous cohorts with one another, and using a non‐Indigenous cohort would omit differences between Indigenous and non‐Indigenous populations.

## 3 RESULTS

### 3.1 Study selection

**FIGURE 1:**
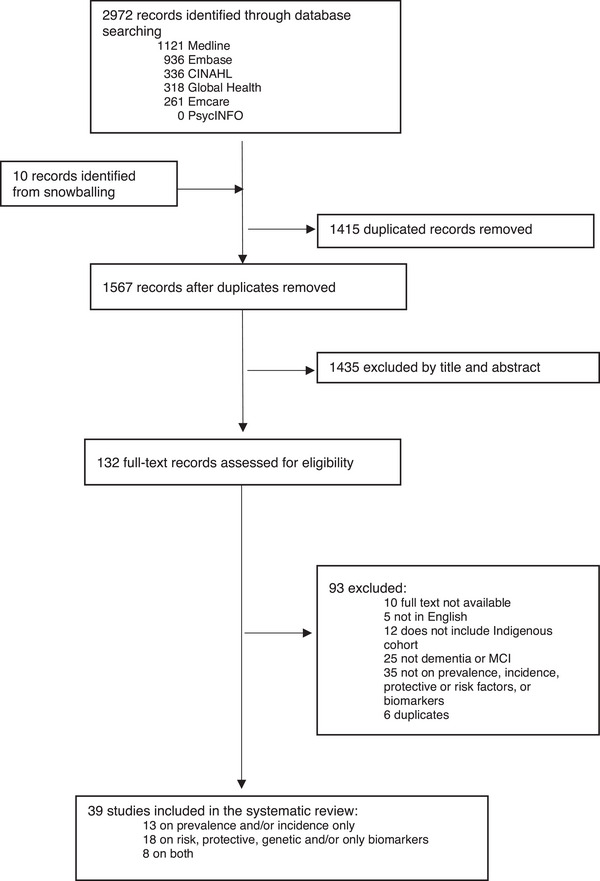
PRISMA flowchart.

Database searches identified 1567 articles after removal of duplications as summarized in the PRISMA flowchart (Figure 1). After screening of titles and abstracts, 132 articles underwent full‐text reviews and 39 met a priori inclusion criteria and were included in this systematic review.

### 3.2 Study characteristics

Of the 39 studies included in this systematic review, 26 studies reported on risk, protective or biomarkers of dementia or cognitive impairment involving an Indigenous population (Table 1). The majority of the included articles were cross‐sectional, case‐control, or cohort studies, and only two involved longitudinal follow‐ups. There were 18 studies on prevalence and 4 on incidence of dementia or MCI (Table 1). Indigenous populations studied included Aboriginal and Torres Strait Islander peoples of Australia, First Nations people of Canada, Choctaw and Cherokee Indians in the United States, Native Hawaiians, Chamorros of Guam, Melanau people of East Malaysia, Tibetans residing in the Qinghai‐Tibet plateau, the Otomi tribe native to Mexico, the Mamirauá and Amanã peoples of the Amazonian Basin in Brazil, and the Tsimane and Moseten tribespeople Indigenous to Bolivia. A Peruvian cohort with high Amerindian genetic ancestry was also included.

**TABLE 1:** Studies included in this systematic review.

	Study	Prevalence and/or incidence	Risk, protective, genetic, and/or biomarkers	Both
Asia				
1.	Pu'un et al., 2014^18^	⬤	⬤	⬤
2.	Huang et al., 2016^19^	⬤	⬤	⬤
3.	Chen et al., 2021^20^		⬤	
Australia & Oceania				
4.	Smith et al., 2008^21^	⬤		
5.	Smith et al., 2010^22^		⬤	
6.	Cotter et al., 2012^23^	⬤		
7.	Li et al., 2014^24^	⬤		
8.	Radford et al., 2015^25^	⬤		
9.	Russell et al., 2016^26^	⬤		
10.	LoGiudice et al., 2016^27^	⬤	⬤	⬤
11.	Radford et al., 2017^28^		⬤	
12.	Radford et al., 2019^29^		⬤	
13.	Derrig et al., 2020^30^	⬤	⬤	⬤
14.	Russell et al., 2021^31^	⬤		
15.	Ma'u et al., 2021^32^		⬤	
16.	Russell et al., 2022^33^		⬤	
17.	Thompson et al., 2022^34^		⬤	
18.	Lavrencic et al., 2022^35^	⬤	⬤	⬤
North America				
19.	Hendrie et al., 1993^36^	⬤		
20.	Rosenberg et al., 1996^37^		⬤	
21.	Henderson et al., 2002^38^		⬤	
22.	Borenstein et al., 2007^39^		⬤	
23.	Sundar et al., 2007^40^		⬤	
24.	Galasko et al., 2007^41^	⬤	⬤	⬤
25.	Jervis et al., 2007^42^	⬤		
26.	Weiner et al., 2008^43^		⬤	
27.	Weiner et al., 2009^44^		⬤	
28.	Weiner et al., 2011^45^		⬤	
29.	Jacklin et al., 2012^46^	⬤		
30.	MacDonald et al., 2015^47^		⬤	
31.	Mayeda et al., 2016^48^	⬤		
32.	Kirkpatrick et al., 2019^49^	⬤		
33.	Carty et al., 2020^50^	⬤	⬤	⬤
34.	Smith et al., 2021^51^		⬤	
35.	Suchy‐Dicey et al., 2021^52^		⬤	
South America				
36.	Stryjer et al., 2011^53^	⬤		
37.	Brucki et al., 2014^54^	⬤		
38.	Marca Ysabel et al., 2021^55^		⬤	
39.	Gatz et al., 2022^56^	⬤	⬤	⬤

Odds ratios (ORs) were the most frequent means of assessing the relationship between variables of interest and dementia or cognitive impairment. MacDonald et al. and Ma'u et al. reported on the Population Attributable Fractions (PAF) of modifiable risk factors and their contribution to dementia in an Indigenous Canadian and Maori cohort, respectively.^32^, ^47^ These PAF calculations utilize the strength of each risk factor, its prevalence in a population, and overlap within the same person to estimate the proportional reduction in dementia at a population level if exposure to the risk factor was eliminated.^57^

### 3.3 Quality assessments

Quality assessments were undertaken on studies on risk, protective factors, and biomarkers (Table 2). Fifteen out of 26 studies were assessed to be of “Good” quality, 9 “Fair” quality, and 2 “Poor” quality based on the modified NIH tool. Engagement, governance, or co‐authorship with Indigenous people/communities/organizations was explicit in 17 studies and 8 studies modeled strength‐based or positive outcomes. A summary of quality assessments is provided in the Appendices (Appendix SC).

**TABLE 2:** Descriptive summary of included studies.

Author, year	Title	Study design	Indigenous population	Age; mean/median age (SD)	Outcomes of interest	Risk or protective factors	Genetic or biomarker	QA
Asia								
Pu'un et al., 2014^18^	Dementia among elderly Melanau: A community survey of an Indigenous people in East Malaysia	Cross‐sectional	Melanau of Malaysia *N* = 344	≥60 years; median 70.4 (±6.7)	Dementia	Dementia was associated with age (OR 1.19^a^, 95% CI 1.12–1.27), no education (OR 7.56^a^ 95%CI 1.70–33.57) and multiple cardiovascular illnesses (OR 3.76^a^, 95% CI 1.25–11.28).		Fair
Huang et al., 2016^19^	Lower prevalence of Alzheimer's disease among Tibetans: Association with religious and genetic factors	Cross‐sectional	Tibetans living in the Qinghai‐Tibet plateau *N* = 3974	>60 years	AD	Age groups 75−79 (OR 4.04, 95% CI 1.27–12.80), 80−84 (OR 3.58, 95% CI 0.96–13.35), ≥85 (OR 35.73, 95% CI 9.64–132.42), eating beef (OR 6.17, 95% CI 2.46–15.49), and head trauma (OR 44.62, 95% CI 2.55–782.17) were associated with AD. Kowtow (0.11, 95% CI 0.027–0.44), turning prayer beads (0.20, 95% CI 0.084–0.47), and eating chicken (OR 0.05, 95% CI 0.005–0.52) were negatively associated with AD.	CLU genotypes AA+GA of rs2279590 were associated with AD (OR 4.48, 95% CI 1.07–18.79). Genotypes GG+GC of rs9331888 (OR 0.18, 95% CI 0.038–0.89) and kowtow (OR 0.20, 95% CI 0.046–0.89) were negatively associated with AD. There was no difference in the distribution of *APOE* alleles between those with and without dementia.	Good
Chen et al., 2020^20^	The association between neprilysin gene polymorphisms and Alzheimer's disease in Tibetan populations	Case‐ control	Tibetans *N* = 212	≥50 years; Mean 68.4 years (±8.3) for AD cohort	AD	No significant difference in gender, age (matched), history of hypertension and T2DM between AD and control groups.	No significant correlation between six polymorphisms of NEP gene loci (rs9829757, rs1816558, rs6776185, rs3736187, rs701109, rs989692) and AD. Allele C of NEP gene locus (rs701109) and allele T of gene locus (rs3736187) were associated with males with AD (*p* < 0.05). *APOE* ε4 was not detected in either cases or control groups.	Fair
Australia & Oceania								
Smith et al., 2010^22^	Factors associated with dementia in Aboriginal Australians	Cross‐sectional	Aboriginal Australians *N* = 363	≥ 45 years; mean 60.9 (±11.9)	Dementia	Older age, male gender (OR 3.1, 95% CI 1.4‐6.8), no formal education (OR 2.7, 95% CI 1.1‐6.7), current smoking (OR^a^ 4.5, 95% CI 1.1‐18.6), previous stroke (OR^a^ 17.9, 95% CI 5.9‐49.7), epilepsy (OR^a^ 33.5, 95% CI 4.8‐232.3), head injury (OR^a^ 4.0, 95% CI 1.7‐9.4), poor mobility (OR 13.4^a^, 95% CI 4.1‐43.9), daytime incontinence (OR 116.8^a^, 95% CI 21.9‐622.8), incontinence at night (OR 87.4^a^, 95% CI 18.4‐415.7), any urinary problems including urinary tract infections, incontinence (OR 4.2^a^, 95% CI 1.8‐10.2) and falls (OR 2.7^a^, 95% CI 1.2‐6.1) was associated with dementia.	N/A	Good
LoGiudice et al., 2016^27^	Incidence and predictors of cognitive impairment and dementia in Aboriginal Australians: A follow‐up study of 5 years	Longitudinal cohort	Aboriginal peoples in Australia *N* = 189	≥ 45 years Mean 65.4 (±10.3)	Dementia; MCI	Factors associated with CIND or dementia were age (OR 1.12^a^, 95% CI 1.06−1.17), poor mobility (OR 3.08^a^, 95% CI 1.09, 8.72), head injury (OR 5.22^a^, 95% CI 1.85−14.70), analgesic medication (OR 3.60^a^, 95% CI 1.35−9.62), and BMI (OR 0.90^a^, 95%CI 0.82−0.98). ***** Factors associated with incident MCI or dementia from normal were stroke (OR 9.54^a^, 95% CI 2.39‐38.13), head injury (OR 3.74^a^, 95% CI 1.14‐12.24), analgesic medication (OR13.48^a^, 95% CI 3.16‐57.44), BMI (OR 0.90^a^, 95% CI 0.81‐1.00) and higher SBP (OR 1.03^a^, 95% CI 1.00‐1.06).	N/A	Good
Radford et al., 2017^28^	Childhood stress and adversity is associated with late‐life dementia in Aboriginal Australians	Cross‐sectional	Aboriginal and Torres Strait Islander people of Australia *N* = 296	≥60 years; median 66.6 (±6.3)	Dementia; AD	Each standard deviation increase in CTQ scores were associated with all‐cause dementia (OR 1.70, 95% CI 1.14−2.54) and AD (OR 1.77, 95% CI 1.08−2.91). CTQ scores and dementia remained significant after controlling for depression and anxiety variables (OR 1.61^a^, 95% CI 1.05−2.45). There were no significant associations between CTQ scores and smoking, alcohol abuse, diabetes, or cardiovascular risk factors.	N/A	Good
Radford et al., 2019^29^	Factors associated with the high prevalence of dementia in older Aboriginal Australians	Cross‐sectional	Aboriginal and Torres Strait Islander people of Australia *N* = 336	≥60 years; median 66.6 (±6.3)	Dementia; AD; MCI	Factors associated with all‐cause dementia included age (OR 2.88^a^, 95% CI 1.93−4.31), childhood trauma (OR 1.59^a^, 95% CI 1.06−2.40), unskilled work (OR 2.41^a^, 95% CI 1.12−5.19), stroke (OR 3.35^a^, 95% CI 1.57−7.15), head injury with loss of consciousness (OR 0.58^a^, 95% CI 1.20−5.56), epilepsy (OR 3.17^a^, 95% CI 0.88−11.49), high risk current alcohol use (OR 3.23^a^, 95% CI 1.17‐8.93), depression (OR 1.60^a^, 95% CI 1.08‐2.37), low BMI (OR 3.44, 95% CI 1.38‐8.61), mobility impairment (OR 2.40, 95% CI 1.20‐4.80), ADL impairment (OR 3.98^a^,	N/A	Good
						95% CI 2.66‐5.96), incontinence (OR 5.56, 95% CI 2.63−11.76, hospitalization in the past year (OR 2.09^a^, 95% CI 1.06‐4.14), residing in residential care (47.72, 95% CI 9.76−233.34), living alone (OR 2.13^a^, 95% CI 1.03‐4.43) and loneliness (OR 3.86^a^, 95% CI 1.70‐8.75. Mild physical activity (OR 0.34^a^ 95% CI 0.14‐0.79), moderate physical activity (OR 0.44^a^ 95% CI 0.19‐0.99 and social activities (OR 0.41^a^ 95% CI 0.27‐0.62) were associated with a lower odds of dementia. ***** Age (OR 3.31^a^ 95% 2.01‐5.46), childhood trauma (OR 1.90^a^, 95% CI 1.15‐3.16) and stroke (OR 3.08, 95% CI 1.21‐7.88) were significant independent factors associated with probable or possible AD. Lifetime low risk alcohol consumption was associated with a lower likelihood of AD relative to abstinence (OR 0.22^a^, 95% CI 0.05‐1.00).		
Derrig et al., 2020^30^	Mild cognitive impairment in Aboriginal Australians	Cross‐sectional	Aboriginal and Torres Strait Islander peoples of Australia *N* = 287	≥60 years; Mean 65.9 years (±5.6)	aMCI; non‐aMCI	aMCI was associated with older age (OR 1.68^a^, 95% CI 1.12‐2.53), head injury (OR 3.19^a^, 95% CI 1.35‐7.56), symptoms of depression (OR 1.5^a^2, 95% CI: 1.04‐2.24), and lower blood pressure (OR 0.53^a^, 95% CI: 0.33‐0.86). naMCI was associated with low education (OR = 4.46^a^, 95% CI: 1.53 to 13.05), unskilled work history (OR^a^ = 5.62, 95% CI: 2.07 to 13.90), higher body mass index (OR = 1.99^a^, 95% CI: 1.30 to 3.04), and moderate to severe hearing loss (OR = 2.82^a^, 95% CI: 1.06 to 7.55).	N/A	Good
Ma'u et al., 2021^32^	Differences in the potential for dementia prevention between major ethnic groups within one country: A cross sectional analysis of population attributable fraction of potentially modifiable risk factors in New Zealand	Cross‐sectional	Māori people of New Zealand *N* = 1286	N/A	Dementia	The total weighted PAF for dementia was 51.4% for Maori people. PAF contributions for 12 risk factors were: obesity (7.3%), hearing loss (6.5%), education (5.6%), social isolation (5.1%), hypertension (5.0%), physical inactivity (5.0%), traumatic brain injury (3.5%), smoking (4.3%), depression (4.2%), diabetes (2.4%), air pollution (1.8%), alcohol (0.7%).	N/A	Good
Russell et al., 2022^33^	Factors associated with the increased risk of dementia found in the Torres Strait	Cross‐sectional	Aboriginal and Torres Strait Islander peoples of Australia *N* = 274	≥45 years; Mean 65.1 years (±10.8)	Dementia	Age (OR 1.14^a^, 95% CI 1.09‐1.20), chronic kidney disease (OR 2.77^a^, 95% CI 1.11‐6.91), cerebrovascular disease (OR = 32.47, 95% CI 8.99‐117.31), higher ICIQ score (OR 1.23^a^, 95% CI 1.01‐1.49) were associated with all‐cause dementia (*p* < 0.05). Lower education, poor mobility, hearing impairment, diabetes, dyslipidaemia and pain and falls screening test scores were associated with dementia in univariate analyses but effects were attenuated in multivariable models.		Good
Thompson et al., 2022^34^	Using health check data to understand risks for dementia and cognitive impairment among Torres Strait Islander and Aboriginal peoples in Northern Queensland—A data linkage study	Cohort	Torres Strait Islander and Aboriginal population in Australia	Mean 48.9 (±10.8) years	Dementia; CIND	Increasing age was significantly associated with CIND and Dementia. Education beyond primary school (secondary or further education (RR = 0.38, 95% CI 0.19−0.76) and moderate physical activity of ≥20 min for ≥5 days in the previous 7 days by self‐reports (RR = 0.26, 95% CI 0.13−0.52) were significant as possible protective factors associated with CIND and dementia.	N/A	Good
						Albuminuria, with or without comorbid hypertension, was associated with later risk of CIND and Dementia but did not reach statistical significance, after adjusting for age. Other vascular risk measures including hypertension, diabetes, hyperglycaemia triglycerides, waist circumference, BMI showed inconclusive or had unexpected associations (lower waist circumference and lower triglycerides) with later CIND and dementia risk.		
Lavrencic et al., 2022^35^	Dementia incidence, APOE genotype, and risk factors for cognitive decline in aboriginal Australians: A longitudinal cohort study	Longitudinal cohort	Aboriginal and Torres Strait Islander peoples in Australia *N* = 155	≥60 years; mean 65.7 (±5.7)	Dementia; MCI	Risk factors associated with incident MCI or dementia include older age (OR 2.29^a^, 95% CI 1.42‐3.70), male sex (OR 4.14^a^, 95% CI 0.60‐10.70), unskilled work history (OR 5.09^a^, 95% CI 1.95‐13.26), polypharmacy (OR 3.11^a^, 95% CI 1.17‐8.28) and past smoking (OR^a^ 0.24, 95% CI 0.08‐0.75). Hearing loss, lower education and vision problems were strong risk factors in bivariate models, but effects were attenuated in multivariable models. Protective factors include years of education and being an ex‐smoker (vs. never smoked).	*APOE* ε4 allele frequency was 24%. Homozygous or heterozygous *APOE* ε4 was associated with incident MCI/dementia (OR 3.96, 95% CI 1.25‐12.50).	Good
North America								
Rosenberg et al., 1996^37^	Genetic factors for the development of Alzheimer disease in the Cherokee Indian	Case‐control	Cherokee Indians	>65	AD	N/A	There is an inverse relationship between degree of genetic Cherokee Indian ancestry and representation of AD, independent of *APOE* ε4 allele status. This relationship diminished with increasing age. For a decrease of 10% in Cherokee ancestry, the odds of developing AD are estimated to be nine times greater at age 65 years but only 1.34 times greater at age 80 years.	Poor
Henderson et al., 2002^38^	Apolipoprotein E4 and tau allele frequencies among Choctaw Indians	Case‐control	Choctaw Indians from the USA *N* = 821	mean 75 years for Dementia APOE cohort	Dementia		Lower prevalence of *APOE* ε4 in the Choctaw population compared to Caucasian comparators. Among those who identify > 1/2 Choctaw ancestry the ε4 allele frequency was 6%, about half that of Caucasians. In those with less than or equal to ½ Choctaw ancestry, there was evidence of an association between the *APOE* ε4 genotype and disease (*p* < 0.05) that was consistent with the degree of association seen in Caucasians. Low prevalence of the tau H2 genotype of 6% (1/5^th^ of Caucasian population) but statistical significance limited by low numbers of individuals with dementia.	Poor
Borenstein et al., 2007^39^	Cycad exposure and risk of dementia, MCI, and PDC in the Chamorro population of Guam	Cross‐sectional	Chamorros on Guam *N* = 1984	≥65 years; Mean 79 years (±7.0) for GD cohort	GD; MCI	Picking (OR 1.42^a^, 95% CI 1.05‐1.91), processing (OR 1.57^a^, 95% CI 1.19−2.06) and eating (OR 1.42^a^, 95% CI 1.13‐1.79) fadang in young adulthood were associated with GD. Picking fadang OR 2.87 (95% CI 1.48‐5.56) and eating fadang as a young adult OR 2.18 (95% CI 1.34‐3.55) were associated with PDC. PARPs for GD for picking 0.13 (95% CI 0.03‐0.22), processing 0.12 (95% CI 0.03‐0.22), and eating fadang 0.22 (95% CI 0.07‐0.38). Associations among men were more consistently significant across all outcome groups compared with women in subanalyses by sex. Consumption of fruit bats or exposure to cycad used as a topical medicine were not significant for any of the outcomes. ***** Picking (OR 1.84^a^, 95% CI 1.32‐2.57), processing (OR 1.83^a^, 95% CI 1.34‐2.49), and eating (OR 1.84^a^, 95% CI 1.39‐2.43) fadang in young adulthood were associated with MCI. PAR proportions for MCI for picking 0.26 (95% CI 0.12‐0.40), processing 0.23 (95% CI 0.09‐0.38), and eating fadang 0.28 (95% CI 0.07‐0.50).	*APOE* ε4 cases varied minimally across case groups or controls and was not statistically significant.	Good
Sundar et al., 2007^40^	Two sites in the MAPT region confer genetic risk for Guam ALS/PDC and dementia	Case‐control	Chamorros on Guam *N* = 600	Mean 74.9 (±7.2) years for GD cohort	GD	N/A	MAPT gene SNP 2 (OR 1.61, 95% CI 1.00−2.62), SNP 9 (OR 1.06, 95% CI 0.57−1.97) and SNP 6 and 9 (OR 3.02, 95% CI 1.10−8.25) were associated with GD. No significant evidence of an association was found between *APOE* and GD.	Fair
Galasko et al., 2007^41^	Prevalence of dementia in Chamorros on Guam: Relationship to age, gender, education, and APOE	Cross‐sectional	Chamorros on Guam *N* = 2029	≥65 years; mean 73.8 (±6.0)	GD; MCI	Age (OR 1.11, 95% CI 1.08–1.13) and low education (OR 0.87, 95% CI 0.84–0.90) were associated with dementia. Gender was not significantly associated with dementia or GD after adjusting for age and education. ***** MCI participants (77.4 ± 6.3 years) were older than cognitively normal participants (72.8 ± 5.2 years) with greater ratio of women to men.	*APOE* ε4 frequency was 5.3% for those with all‐cause dementia compared to 4.0% for unaffected controls. *APOE* ε4 was not significantly associated with all‐cause dementia, GD, PDC or vascular dementia.	Good
Weiner et al., 2008^43^	Atherosclerosis risk factors in American Indians with Alzheimer disease: Preliminary findings	Case‐control	American Indians from the United States *N* = 68	Median 78 (range 56‐91) years for AD cases	AD	There is an association between history of hypertension and diabetes and AD in a small sample of American Indians. A high percentage of Choctaw cases and controls had a history of hypertension compared to white participants. History of diabetes was most prevalent in Indian ADs; less so in Indian controls and White participants with AD. Age, education, waist size, BMI, history of high cholesterol, history of myocardial infarction or history of stroke did not differ significantly between the three groups.	Plasma homocysteine concentrations increased with age (*p* < 0.001) but was not significantly associated with degree of Indian heritage (*p* = 0.63); sex (*p* = 0.14) or AD (*p* = 0.14).	Fair
Weiner et al. 2009^44^	Brain MRI, apolipoprotein E genotype, and plasma homocysteine in American Indian Alzheimer disease patients and Indian controls	Case‐control	American Indians from the United States *N* = 21	>60 years	AD	No statistically significant differences between AD subjects and controls in age, gender, degree of Indian heritage, history of hypertension, diabetes, high cholesterol, waist circumference or BMI. Education was lower in the AD group but this was not statistically significant.	More AD subjects had *APOE* ε4 alleles than controls (*p* = 0.043) but there was no significant relationship between the presence of an APOEε4 allele and Indian heritage in the AD group or in the combined AD and control groups. Median plasma homocysteine concentrations were higher in AD subjects but did not achieve statistical significance. There were no significant differences in neuroimaging findings between the two groups, but AD subjects had greater volume of WMH and greater WMHV/WBV ratio (median 1.63% vs. 0.65%) and a far greater range of WMHV. In those with AD, WBV correlated with BMI (*p* = 0.015) and age (*p* = 0.005).	Fair
Weiner et al., 2011^45^	The relationship of cardiovascular risk factors to Alzheimer disease in Choctaw Indians	Case‐control	American Indians from the United States *N* = 78	Median 78 years (range 54‐91) years for AD cases	AD	There were no significant differences between Choctaws with and without AD in gender, number or types of cardiovascular risk factors (history of hypertension, diabetes, high cholesterol and myocardial infarction). Indian AD groups had a significantly higher percentage of affected first‐degree relatives than the Indian controls.	Choctaw groups with and without AD had lower proportion of one or more *APOE* ε4 alleles than White participants. There was a significant relationship between homocysteine concentration and onset age for American Indians with AD (*p* = 0.005).	Fair
MacDonald et al., 2015^47^	Implications of risk factors for Alzheimer's disease in Canada's Indigenous population	Cohort	Indigenous peoples of Canada (First Nation, Inuit, Metis)	N/A	Dementia	Physical inactivity (32.5%, 95% CI 10.1%−51.1%), low educational attainment (22.4%, 95% CI 14.6%−29.6%), smoking (19.4%, 95% CI 5.8%−32.9%), midlife obesity (16.8%, 95% CI 10.3‐23.6), midlife hypertension (14.2%, 95% CI 4.2%−25.1%) and diabetes mellitus (6.0%, 95% CI 2.6%−9.7%) contribute to dementia rates in the Indigenous population. The combined PAR for AD for all six modifiable risk factors was 79.6% among on‐reserve Indigenous, 74.9% among off‐reserve Indigenous, and 67.1% among non‐Indigenous peoples in Canada.	N/A	Good
Carty et al. 2020^50^	Risk factors for Alzheimer's disease and related Dementia diagnoses in American Indians	Cohort	American Indians *N* = 3464	≥55 years	Dementia	Age (RR 1.12^a^ 95% CI 1.11‐1.14), hypertension (RR 2.75^a^ 95% CI 1.75‐4.31), depression (RR 2.49^a^ 95% CI 1.79‐3.46), hyperlipidaemia (RR 1.41^a^ 95% CI 1.08‐1.84) and diabetes (RR 2.07^a^ 95% CI 1.41‐3.03) were associated with increased risk of a dementia diagnosis. Female sex (RR 0.70^a^ 95% CI 0.52‐0.94), being married/having a life partner (RR 0.67^a^ 95% CI 0.48‐0.94) were associated with lower risk of a dementia diagnosis.		Fair
Smith M et al., 2021^51^	Disparities in Alzheimer disease and mild cognitive impairment among Native Hawaiians and Pacific Islanders	Cohort	Native Hawaiians & Pacific Islanders *N* = 87	N/A	AD; MCI	The mean age of Native Hawaiians and Pacific Islanders at time of diagnosis with AD or MCI was 73.2 (±12.5) years. Mean age at diagnosis was lower in this group with greater percentage with a diagnosis <65 years compared to other racial groups (*p* = 0.02). There was a higher proportion of women than men with diagnosis of AD over MCI 67%, *p* = .028). Hypertension, hyperlipidaemia, and type II diabetes were found to be higher among the Native Hawaiians and Pacific Islanders (hypertension 74%, *p* = 0.012; hyperlipidemia 70%, *p* =0 .05; T2DM 28%, *p* = 0.002). There were no statistically significant differences in marital status, insurance and number of comorbidities between the different racial groups.		Fair
Suchy‐Dicey et al., 2021^52^	APOE genotype, hippocampus, and cognitive markers of Alzheimer's disease in American Indians: Data from the Strong Heart Study	Cohort	American Indians *N* = 811	≥64 years Mean 73.1 (±6.0)	Cognitive impairment^b^		APOE ε4 carrier frequencies were 4/3 21.0%; ε4/4 0.7%; ε4/2 0.6% (total 22.3%). Non‐APOE ε4 carrier frequencies include ε3/3 72.5%; ε3/2 5.1%; ε2/2 0%. Sociodemographic and clinical features were similar between APOE ε4 carriers and non‐carriers apart from higher proportion with LDL and chronic kidney disease (not significant). MRI‐defined brain volumes (brain, hippocampal, intracranial volumes) and multidomain cognitive test scores were similar between APOE ε4 carriers and non‐carriers and not significantly associated with carrier status, after adjusting for sociodemographic and clinical conditions.	Good
South America								
Marca Ysabel 2021^55^	Dissecting the role of Amerindian genetic ancestry and the *APOE* ε4 allele on Alzheimer disease in an admixed Peruvian population	Case‐control	Peruvian *N* = 207	≥65 years	AD	N/A	Genetic ancestry surrounding the *APOE* is predominately Amerindian (60.6%). *APOE* ε4 allele frequency was 9.2% in cognitively impaired individuals versus 4.6% in those cognitively normal and significantly associated with increased risk of AD (OR 5.02^a^, 95% CI 2.3−12.5)	Fair
Gatz et al., 2022^56^	Prevalence of dementia and mild cognitive impairment in Indigenous Bolivian forger‐horticulturalist	Cohort	Indigenous Tsimane and Moseten people of Bolivia *N* = 623	≥60 years	Dementia; MCI	Dementia cases were equal between women and men. ***** MCI was more prevalent among women compared to men (*p* = 0.02 for sex difference). ***** Cognitive impairment was associated with visuospatial impairments, parkinsonian symptoms and gait abnormalities.	The number of *APOE* ε4 alleles did not distinguish between those with cognitive impairment and those without however, carrying two (vs. none) alleles was associated with significantly greater odds of cognitive impairment (Tsimane OR 10.7 95% CI 1.47, 78.6; Moseten OR 11.5 95% CI 1.28‐102.8). On CT, medial temporal atrophy (OR 8.89 95% CI 1.85‐42.9), internal carotid artery calcification morphology (OR 6.10 95% CI 1.23‐30.4), basal ganglia calcification (OR 5.27 95% CI 1.85‐∞), calcification of the lenticulostriate arteries (OR 4.77 95% CI 1.04‐22.0) and lenticulostriate arteries calcification density (OR 1.11 95% CI 1.01‐1.23) was associated with cognitive impairment for Tsimane but not for Moseten. Poorer cognition was associated with severity of intracranial vascular calcification, greater severity of cerebral atrophy, especially of white matter volume.	Good

Abbreviations: AD, Alzheimer's dementia; aMCI, amnestic mild cognitive impairment; APOE4, apolipoprotein E allele; BMI, body mass index; CIND, cognitive impairment not dementia; CLU, clusterin; CT, computed tomography; CTQ, Childhood Trauma Questionnaire; DBP, diastolic blood pressure; eGFR, estimated glomerular filtration rate; Fhx dementia, family history of dementia; GD, Guam dementia; ICIQ, International Consultation on Incontinence Questionnaire; MAPT, microtubule‐associated protein tau; MCI, mild cognitive impairment; MRI, magnetic resonance imaging; NEP, neprilysin; OR, odds ratio; PAF, population attributable fractions; PAR, population attributable risk; PDC, parkinsonism dementia complex; PTSD, post‐traumatic stress disorder; QA, quality assessment; RICE, Retrospective Indigenous Childhood Enrichment Scale; RR, relative risk; SBP, systolic blood pressure; T2DM, type 2 diabetes mellitus; WHMH, white matter hyperintensities; WHV, white matter hyperintensities volume.

^a^ Adjusted/multivariate model.

^b^ Cognitive impairment was defined as deficits (1.5 SD below mean) in leaning and retention tasks in multidomain psychometric tests such as the California Verbal Learning Test, Weschler Adult Intelligence Scale, Controlled Oral Word Association, and Modified Mini Mental Status Examination.

### 3.4 Prevalence and incidence of dementia and MCI

Most studies included participants aged ≥60 (±5 years). For those studies with participants aged ≥45 years, prevalence rates for ≥60 years and age‐standardized prevalence rates are reported where available. Across[Table alz13458-tbl-0003] different Indigenous populations, prevalence rates of dementia ranged from 0.6% to 37.2% and rates of MCI/CIND varied from 4.4% to 27.4%. Low rates of dementia were reported for Cree Indians (0.5%), Tibetans (1.3%), Tsimane (1.2%), and Moseten (0.6%) cohorts^19^, ^36^, ^56^ and highest for Aboriginal and Torres Strait Islander peoples (up to 26.8% in those ≥65 years).^21^ Dementia incidence reported for Aboriginal and Torres Strait Islander peoples ranged from 21.0 to 35.9 per 1000 person‐years for those aged ≥60 years.^22^, ^25^ Mayeda et al.^48^ reported a dementia incidence of 22.2 per 1000 person‐years for American Indians ≥60 years (Table 3).

**TABLE 3 alz13458-tbl-0003:** Prevalence and incidence.

		Indigenous population sample size	Age; mean/median age (SD)	Outcome:	
Study	Indigenous population			Prevalence	Incidence
**Asia**					
Pu'un et al., 2014^18^†	Melanau of East Malaysia	344	≥60 years; mean 70.4 (±6.7)	Dementia 10.5%; Any cognitive impairment 27.3%	
Huang et al., 2016^19^†	Tibetans from the Qinghai‐Tibet plateau	3974	≥60 years	Dementia 1.3% (95% CI 0.9−1.7)	
**Australia & Oceania**					
Smith et al., 2008^21^	Aboriginal and Torres Strait Islander peoples Kimberley region of WA, Australia	363	≥45 years; mean 60.7 (±11.9)	Dementia 12.4% (95% CI 9.0‐15.8); 26.8% (95% CI 18.8‐34.8) ≥65 years CIND of 8.0%; 13.4% ≥65 years	
Cotter et al., 2012^23^	Aboriginal and Torres Strait Islander peoples, Australia	1668 (multiple data sources)	≥45 years	Dementia 0.4–6.3 per 1000 persons	
Li et al., 2014^24^	Aboriginal and Torres Strait Islander peoples from the Northern Territory, Australia	11,646 (multiple data sources)	≥45 years; median 72	Dementia 3.7%; 6.5% (95% CI 5.8‐6.8)*	Dementia 27.3 cases per 1000 person‐years (95% CI 22.8‐31.8)*
Radford et al., 2015^25^	Aboriginal and Torres Strait Islander peoples from New South Wales, Australia	336	≥60 years; median 66.6 (±6.3)	Dementia 13.4% (95% CI 9.6‐17.2); 21.0% (95% CI 12.8‐29.2)* MCI 17.7% (95% CI 3.4‐21.9)	
Russell et al., 2016^26^	Torres Strait Islanders from the Torres Strait, Australia	20	≥ 45 years; mean 65.8 (±9.5)	Dementia 5.0% CIND 20.0%	
LoGiudice et al., 2016^27^†	Aboriginal and Torres Strait Islander peoples Kimberley region of WA, Australia	189	≥45 years; mean 65.4 (±10.3)		Dementia: 7.3 (95% CI 3.7‐14.5) per 1000 person‐years; 21.0 (95% CI 10.5‐42.1) per 1000 person years for ≥60 years MCI or dementia 28.5 (95% CI 20.0‐40.5) per 1000 person‐years; 52.6 (95% CI 33.9, 81.5) per 1000 person‐years for ≥60 years
Derrig et al., 2020^30^†	Aboriginal and Torres Strait Islander peoples from New South Wales, Australia	287	≥60 years; mean 65.9 (±5.6)	MCI 18.4%	
Russell et al., 2021^31^	Torres Strait Islanders from the Torres Strait, Australia	274	≥45 years; mean 65.1 (±10.8)	Dementia 14.2% CIND 21.9%	
Lavrencic et al., 2022^35^†	Aboriginal and Torres Strait Islander peoples from New South Wales, Australia	155	≥60 years; mean 65.7 (±5.7)		Dementia: 35.9 per 1000 person‐years (95% CI 18.3‐53.6)* MCI and dementia: 75.2 (95% CI 50.6‐99.8)*
**North America**					
Hendrie et al., 1993^36^	The Cree of Manitoba, Canada	192	≥65 years	Dementia 4.2% AD 0.5%	
Galasko et al., 2007^41^†	Chamorros of Guam, United States	1984	≥65 years; mean 73.8 (±6.0)	Dementia 12.2% (95% CI 11.7‐12.9) GD/AD 8.8% (95% CI 8.3‐9.4) MCI 4.4%	
Jervis et al., 2007	American Indians, United States	140	≥60 years; mean 69.8 (±6.4)	Dementia 14.6% based on the mini‐Mental State Examination; 37.2% based on the Mattis Dementia Rating Scale CIND 27.4% for MDRS; 13.4% for >65	
Jacklin et al., 2012^46^	First Nations people of Alberta, Canada	129 774	N/A	Dementia 7.5(95% CI 6.6‐8.5) per 1000*	
Mayeda et al., 2016^48^	American Indians and Alaska Natives, United States	4543	≥60 years; mean 73.4		Dementia 22.2 (95% CI 20.9–23.5) per 1000 person
Kirkpatrick et al., 2019^49^	American Indians, United States	52	Mean 64 (±7.1)	44.2% (95% CI 30‐50) with cognitive impairment based on the Montreal Cognitive Assessment 15 participants underwent further evaluation: non‐amnestic MCI (27%); vascular MCI (33%); vascular dementia (13%)	
Carty et al. 2020^50^†	American Indians, United States	3464	≥55 years, median 64	Dementia 5.7%; 6.6%*	
**South America**					
Stryjer et al., 2011^53^	Otomis tribe from Queretaro, Mexico	65	≥65 years; mean 73.3 (±7.7)	Dementia 12.3% based on the diagnostic and statistical manual of mental disorders‐fourth edition (DSM‐IV); 7.7% based on Brookdale test and 6.1% with both	
Brucki et al., 2014^54^	Mamirauá and Amanã peoples of the Amazonian Basin, Brazil	163	≥50 years; mean 62.3 (±9.2)	Dementia 4.9%; 12.3 ≥65 years CIND 6.1%; 7.7% for ≥65 years	
Gatz et al., 2022^56^†	Indigenous peoples of Bolivia	623	≥60 years	Dementia in Tsimane 1.2% (95% CI 0.4‐2.7) ; 2.7% (95% CI 0.1‐5.4)*; in Moseten 0.6% (95% CI 0.0‐3.2) ; 0.9%* (95% CI 0.0‐2.6)* MCI in Tsimane 7.7% (95% CI 5.2‐10.3)*; in Moseten 9.8% (95% CI: 4.9, 14.6)*	

Abbreviations: AD, Alzheimer's dementia; CI, confidence interval; CIND, cognitive impairment not dementia; GD, Guam dementia; MCI, mild cognitive impairment; N/A, not applicable; SD, standard deviation.

*Age‐adjusted values.

†Articles included in Table 2.

### 3.5 Risk and protective factors

Modifiable risk factors featured across multiple (>1) studies that demonstrated a statistically significant association include childhood adversity, hearing loss, low education attainment, unskilled work history, stroke, head injury, epilepsy, diabetes, hypertension, hyperlipidemia, depression, low BMI, poor mobility, and continence issues by self‐reports or using the International Consultation on Incontinence Questionnaire score. Non‐modifiable risk factors across different studies were increasing age, sex, and genetic polymorphisms, including *APOE* ɛ4. Age was reported to be statistically significant in 11 studies, and sex differences were reported in 7 studies. Gene associations with dementia or MCI were highlighted in 13 studies (Table 4).

**TABLE 4:** Compilation of studied risk factors with measures of association.

Studied variable of interest	Pu'un, 2014^18^	Huang, 2016^19^	Chen, 2020^20^	Smith, 2010^22^	LoGiudice, 2016^27^	Radford 2017^28^	Radford, 2019^29^	Derrig, 2020^30^	Russell, 2022^33^	Thompson 2022^34^	Lavrencic, 2022^35^	Rosenberg, 1996^37^	Henderson, 2002^38^	Bronstein, 2007^39^	Sundar 2007^40^	Galasko, 2007^41^	Weiner, 2008^43^	Weiner, 2009^44^	Weiner, 2011^45^	Carty et al., 2020^50^	Smith et al., 2020^51^	Suchy‐Dicey et al., 2022^52^	Marca Ysabel 2021^55^	Gatz, 2022^56^
Region	Asia			Australia & Oceania								North America											South America	
**NON‐MODIFIABLE**																								
Age								^*^																
Sex				**M**							**M**			**M**		**M**				**F**	**F**			^*^ **F**
Family history of dementia																								
Ethnicity																								
APOE4		**NS**												**NS**	**NS**	**NS**			**NS**			**NS**		
Tau													**NS**											
NEP			**M**																					
CLU																								
MAPT																								
Homocysteine																	**NS**	**NS**						
**POTENTIALLY MODIFIABLE**																								
Childhood trauma/adversity																								
Religious practices																								
Education								^*^																
Unskilled work history								^*^																
Social activities																								
Physical activity																								
Hospitalisation in past year																								
Married/life partner																								
Living alone																								
Living in residential care																								
Loneliness																								
Diet: beef																								
Diet: chicken																								
Exposure to cyad‐derived products																								
Smoking											^**^													
Alcohol																								
Stroke																								
Head injury																								
Epilepsy																								
Cardiovascular disease																								
Renal disease/Albuminuria																								
Obesity/high BMI								^*^																
low BMI																								
Hyperlipidaemia																								
Depression/PTSD								^*^																
Diabetes/HbA1c																								
Hypertension/high BP																								
Low BP								^*^																
Hearing loss								^*^																
Vision impairment																								
Analgesics																								
Polypharmacy																								
Mobility impairment																								
Falls																								
Urinary incontinence																								
ADL impairment																								

*Note*: Blue indicates a statistically significant negative association. Green indicates a statistically significant positive association. NS indicates not statistically significant.

Abbreviations: ADL, activities of daily living; APOE4, apolipoprotein E allele; BMI, body mass index; CLU, clusterin; DBP, diastolic blood pressure; eGFR, estimated glomerular filtration rate; F, female; HbA1c, hemoglobin A1C; M, male; MAPT, microtubule‐associated protein tau; NEP, neprilysin; PTSD, post‐traumatic stress disorder; SBP, systolic blood pressure.

* Mild cognitive impairment/cognitive impairment not dementia only.

** Ex‐smoker compared to never smoked.

Potential protective factors or those negatively associated with the occurrence of dementia or MCI reported to be statistically significant in at least one study include: higher education attainment, ex‐smoking (compared to never smoked or current smoker), Kowtow, turning prayer beads, physical activity by self‐reports, social activities, being married or having a life partner, and eating chicken (Table 4). Low BMI was reported to be associated with a greater odds of dementia and cognitive impairment in two studies^27^, ^29^ but was associated with a lower odds of MCI in one study^30^ involving Aboriginal and Torres Strait Islander peoples. Seven out of nine studies that highlighted protective factors were assessed to be of good quality,^19^, ^22^, ^28^, ^30^, ^34^, ^35^, ^41^ and two were of fair quality^18^, ^50^ (Table 2).

PAF were reported in three studies.^32^, ^39^, ^47^ MacDonald et al. produced a combined PAF for AD of 79.6% and 74.9% among on‐reserve and off‐reserve Indigenous peoples in Canada, respectively. The relative contributions of six modifiable risk factors were physical inactivity (32.5%), low education attainment (22.4%), smoking (19.4%), midlife obesity (16.8%) midlife hypertension (14.2%), and diabetes mellitus (6.0%).^47^ Ma'u et al. illustrated a PAF of 51.4% using 12 risk factors including obesity (7.3%), hearing loss (6.5%), education (5.6%), social isolation (5.1%), hypertension (5.0%), physical inactivity (5.0%), traumatic brain injury (3.5%), smoking (4.3%), depression (4.2%), diabetes (2.4%), air pollution (1.8%), and alcohol (0.7%) for Māori people of New Zealand.^32^ Exposure to fadang in the unique population of Chamorros on Guam was estimated to contribute between 12% and 23% to outcomes of Guam dementia and MCI, after adjusting for age, sex, and education.^39^

### 3.6 Genetic markers

Apolipoprotein E allele4 (*APOE* ε4) was the most studied genetic marker and featured in 12 studies. Half of these studies found no association between the distribution of *APOE* alleles between participants with and without dementia.^19^, ^39^, ^40^, ^41^, ^45^, ^52^ *APOE* ɛ4 allele frequency was reported in a study of Aboriginal and Torres Strait Islander peoples (24%), Chamorros (5.3% in dementia vs. 4.0% in cognitively normal group), American Indians (22.3%), and Peruvians with American Indian ancestry (9.2% in cognitively impaired vs. 4.6% in cognitively normal group).^35^, ^41^, ^52^, ^55^ Greater odds of MCI and dementia associated with being a homozygous or heterozygous carrier of the *APOE* ɛ4 allele (OR 3.96, 95% 1.25‐12.50) was described in one longitudinal cohort of Aboriginal and Torres Strait Islander peoples.^35^ Marca‐Ysabel et al. found *APOE* ɛ4 to be associated with increased risk of AD (OR 5.02, 95% CI 2.3‐12.5).^55^ Among the Indigenous Tsimane and Moseten peoples, the number of *APOE* ɛ4 alleles did not differ between those with cognitive impairment and those without; however, carrying two *APOE* ɛ4 alleles was associated with greater odds of cognitive impairment.^56^ A couple of studies looked at the relationship between *APOE* ɛ4, degree of American Indian ancestry and dementia. Henderson et al. found that the prevalence of *APOE* ɛ4 in the Choctaw population was half that of Caucasian comparators. For those with less than 50% Choctaw ancestry, there was evidence of an association between the *APOE* ɛ4 genotype and disease.^38^ However, Rosenberg reported an inverse relationship between the genetic degree of Cherokee ancestry and AD, independent of *APOE* ɛ4 status.^37^

Other genes studied include clusterin (CLU), neprilysin (NEP), microtubule‐associated protein tau (MAPT) and Tau.^19^, ^20^, ^40^ Huang et al. reported that CLU genotypes AA and GA of rs2279590 were associated with a 4‐fold increase in the odds of AD while CLU genotypes GG and GC of rs9331888 were negatively associated with AD in a sub‐sample of 39 Tibetans with AD and 56 without.^19^ Chen et al. reported two different NEP SNPs (rs701109 and rs3736187 mutation) that may add to the risk of AD among male Tibetans rather than the whole population.^20^ Sundar et al. reported MAPT SNPs 6, 7, and 9, homozygous genotypes (A/A, A/A, and C/C, respectively) contribute to susceptibility to Guam neurologic disorders.^40^ Those with SNP6 and SNP9 AC/AC diplotype had a three‐fold increased risk for Guam dementia and a 4‐fold increased risk for Parkinsonism dementia complex compared to those with other diplotypes, after adjusting for SNP2. SNP2 allele carriers had a 1.6‐fold increased risk of Guam dementia and a 2‐fold increased risk of Parkinsonism dementia complex, after adjusting for SNPs 6 and 9.^40^

### 3.7 Other biological and neuroradiological markers

Plasma homocysteine was a biological marker found to be higher in a small group of American Indians with AD compared to controls but did not reach statistical significance.^43^, ^44^

There were only three studies that reported on neuroimaging markers related to dementia and MCI, one using CT imaging and two using MRI.^44^, ^52^, ^56^ There were no PET imaging or cerebrospinal fluid biomarker studies. In the Bolivian cohort, poorer cognition was associated with severity of intracranial vascular calcifications, greater medial temporal atrophy, and reduced white matter volume on CT brain scans of Tsimane participants.^56^ In a small case‐control study of 21 participants by Weiner et al., AD participants demonstrated greater volume of white matter hyperintensity as a proportion of whole brain volumes on MRI, but this did not reach statistical significance.^44^ Suchy‐Dicey et al. described MRI‐defined brain, hippocampal, and intracranial volumes against cognitive test scores in a larger cohort of 811 American Indians but found no significant association between neuroradiological parameters between *APOE* ɛ4 carriers and non‐carriers after adjusting for sociodemographic and clinical conditions.^52^

## 4 DISCUSSION

### 4.1 Availability of evidence across Indigenous groups

**FIGURE 2:**
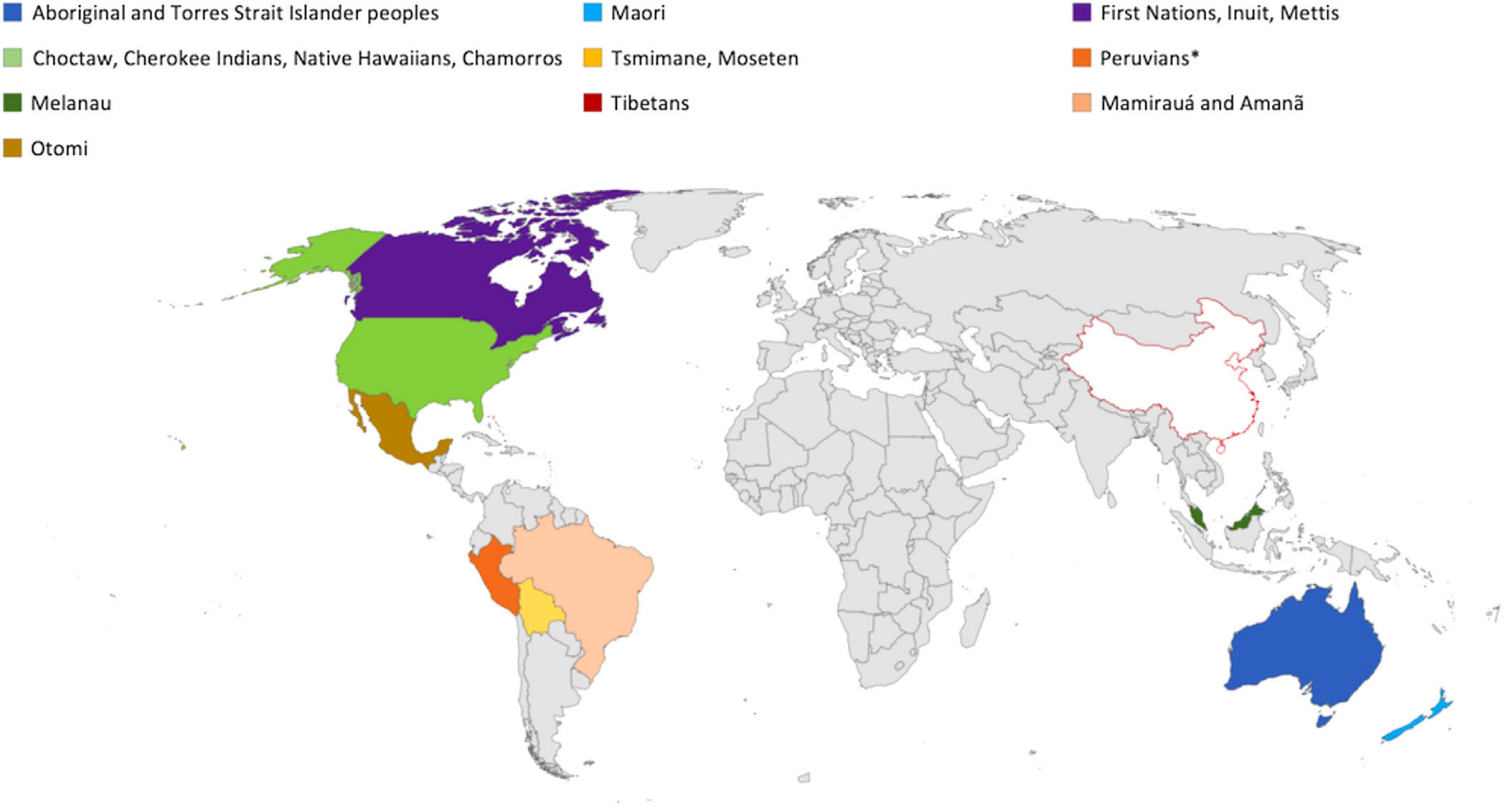
Geographical representation of Indigenous peoples included in this systematic review.

Indigenous peoples live in every corner of the globe with the largest populations expected to reside in populous China, India, and Siberian regions of Russia.^58^ However, most of the researched Indigenous groups included in this systematic review are from Western countries with a paucity of published scientific literature from these regions (Figure 2). Compared to most world regions where age‐standardized prevalence of dementia in those 60 and older lie between 5% and 7%, there was greater variance in the reported prevalence rates within and between Indigenous populations.^59^ Prevalence rates were lowest for Cree Indians, Tibetans, Tsimane, and Moseten (0.5%–1.3%) and highest among Aboriginal and Torres Strait Islander peoples (>20%) in those ≥60 years.^19^, ^21^, ^25^, ^36^, ^56^ Age‐adjusted dementia rates for American Indians ranged from 6.6% to 26.8% between studies, compared to the estimated prevalence of 10.8% in the general United States population.^60^ This may reflect the heterogeneity of study methodologies. For instance, the study by Cotter et al.^23^ based on databases reliant on routinely collected data reported no difference in dementia rates between Aboriginal and Torres Strait Islander peoples and non‐Indigenous Australians, which was not congruent with cohort studies based on expert clinical diagnoses of dementia.^21^, ^25^, ^31^ The latter consistently report rates of dementia three to five times greater among different cohorts of Aboriginal and Torres Strait Islander peoples compared to non‐Indigenous Australians and are more likely to reflect true prevalence of disease.^21^, ^25^, ^31^

### 4.2 Evidence of risk and protective factors of dementia or cognitive impairment

Despite the diversity of Indigenous peoples worldwide, there are many similarities in the determinants of health and illness. Study of risk factors predominate the literature and emphasize medical factors such as head injury, epilepsy, diabetes, hypertension, and stroke. However, protective factors associated with dementia or MCI are less well understood. Poor mobility, falls, and continence issues found to be associated with dementia in cross‐sectional studies likely reflect syndromes related to a dementia diagnosis rather than its precipitant. This may have a bearing on the association between BMI and dementia across different studies. This relationship is proposed to be J‐shaped, whereby high BMI and obesity in middle age may increase the risk of dementia but low BMI is more likely to accompany cognitive impairment or dementia in the older population.^61^

Risk and protective factors associated with dementia for Indigenous peoples are congruent with the evidence available for non‐Indigenous populations.^4^, ^57^ However, the significance of social determinants of health across the life‐course (childhood adversity, education attainment, skilled work history) and engagement with cultural or religious practices (Kowtow, turning prayer beads) were highlighted in this review of Indigenous peoples. As Radford et al. proposed, cultural connection and support from extended family may help mitigate experiences of trauma and adversity in childhood.^28^ Male sex was associated with a greater risk of dementia in certain Indigenous cohorts,^22^, ^35^, ^39^, ^41^, ^46^ in contrast to most non‐Indigenous populations where dementia is more common in women.^62^, ^63^ Higher rates of cluster risk factors such as stroke, head injury, and cardiac disease among men compared to women have been described in studies involving Aboriginal and Torres Strait Islander peoples.^22^, ^29^, ^35^ Sex differences in exposure to other lifestyle and social risk factors such as heavy alcohol use, history of police custody and incarceration, and low education may also contribute to the higher rates of dementia and cognitive impairment among men in these communities. Among the Chamorros, there was slightly higher prevalence of parkinsonism‐dementia complex in men, but sex was not significantly associated with Guam dementia after adjusting for age and education.^39^, ^41^ Studies involving First Nations people of North America included in this review were equivocal and contained insufficient data on rates of risk factors to offer plausible explanations on sex differences. More work is required to delineate the differential risk factors for dementia for Indigenous men and women and to ascertain whether adjustments for education, cardiometabolic disease, and competing risk of death modifies these sex differences.^64^

PAF calculations utilize the strength of each risk factor, its prevalence in a population, and overlap within the same person. Combined PAF for AD for First Nations people of Canada (79.6% for on‐reserve and 74.9% for off‐reserve) and Māori (51.4%) were considerably higher than global estimates (40%) and suggests that the preventable burden of dementia may be greater for certain Indigenous communities.^4^, ^32^, ^47^, ^57^ This is in keeping with the breadth of studies demonstrating higher prevalence of health and psychosocial factors associated with dementia in different Indigenous, compared to non‐Indigenous, populations.^2^

### 4.3 Genetic factors associated with dementia or cognitive impairment

*APOE* ɛ4 is the most well recognized genetic risk‐factor for AD. The association of *APOE* allele with AD risk differs across ethnoracial groups with stronger effects observed in East Asian (ε3/ε4 OR: 3.1‐5.6; ε4/ε4 OR: 11.8‐33.1) and non‐Hispanic White populations (ε3/ε4 OR: 3.2; ε4/ε4 OR: 14.9) compared to African Americans and Hispanic peoples (ε3/ε4 OR:1.1‐2.2; ε4/ε4 OR: 2.2–5.7).^65^ Most studies of *APOE* ɛ4 involving Indigenous peoples were small and may be poorly powered to discern an association. Where an association was reported, *APOE* ɛ4 carriers had increased odds of dementia or cognitive impairment comparable to other high‐risk ethnic groups, except among American Indians where the degree of Choctaw or Cherokee ancestry may modify this effect. The genotype ɛ2/ɛ3 that has been demonstrated to be protective across ethnic groups has not demonstrated in studies involving Indigenous peoples.^65^

Genome‐wide association studies involving Indigenous peoples are limited and more work is required to elucidate genetic polymorphisms of significance to individual populations and across different cohorts. There was a notable absence of studies on AD susceptibility genes such as amyloid precursor protein, presenilin, presenilin 2, phosphatidylinositol‐binding clathrin assembly protein, or inflammatory markers (e.g., C‐reactive protein, interleukin‐6) that have been the subject of dementia research in non‐Indigenous populations.^66^

### 4.4 Neuroradiological and biomarkers for dementia and cognitive impairment

This systematic review yielded few studies involving biological or imaging markers of dementia or MCI. Many of these studies were small scale, case‐control studies with limited statistical power.^44^, ^56^ In the broader literature, a study of 786 American Indian participants from the Strong Heart study (not included in this review) found that general cognitive functioning correlated with hippocampal volumes while processing speed was associated with brain volumes as well as cerebral vascular burden (infarcts and white matter disease).^67^ In studies comparing ethnoracially diverse groups, cognitive decline was most associated with global gray matter atrophy in African American participants, volume of white matter hyperintensities in Hispanic participants, while regional temporal atrophy had greater relevance for White American participants.^68^ This suggests t differences in the importance of neuroimaging markers as a reflection of the heterogenous brain changes driving cognitive change and dementia between ethnoracial groups. Larger studies on neuroimaging markers of dementia and MCI involving Indigenous peoples are needed to greater understand the brain changes relevant to these populations.

### 4.5 Indigenous perspectives in dementia research

The dementia prevention discourse is largely framed by a Western biomedical model with emphasis on risk factors and pathology.^69^ Though this model allows for greater recognition of those at risk of cognitive decline and dementia, it often de‐emphasizes the cultural, historic, and psychosocial influences that are crucial in comprehensive understanding of possible determinants and correlates of health for Indigenous peoples. This biomedical model also reinforces a deficits approach that can negatively impact on the empowerment of people to strive for healthy ageing. Alternatively, a greater focus on proactive factors across the life‐course may be more conducive to a strengths‐based approach and the promotion of cognitive health and ageing for Indigenous peoples and their communities. There is increasing awareness of the Ethics around research involving Indigenous peoples evident in approaches to study design and governance of research teams. Some research groups are explicit in their engagement of Indigenous researchers and stakeholders to ensure that research involving Indigenous people serves to strengthen capacity, improve health outcomes and progress interests valued by Indigenous peoples and communities.^70^

### 4.6 Future directions

Increased research is needed on *modifiable protective factors* of dementia involving Indigenous peoples to develop targeted *strength‐based models* that promote cognitive health and prevent impairment *over the life‐course*. The absence of studies on disease‐specific biomarkers of dementia or cognitive impairment involving an Indigenous cohort in this review highlights a significant research gap that is out of keeping with worldwide efforts to validate dementia biomarkers and to see to their incorporation into diagnostic and classifications schemes in research and clinical settings.^71^, ^72^ Greater efforts to engage Indigenous peoples in *biomarker studies and clinical trials* in *culturally safe ways* are needed to harness the potential to translate research to improve dementia diagnosis and care in the future. Research should be conducted with *explicit partnership* with Indigenous peoples and communities.

## 5 CONCLUSIONS

There are many shared determinants of health and illness across Indigenous people, but risk profiles may differ between populations. Greater understanding of modifiable risk and protective factors, backed by validated disease‐specific biomarkers, would be useful in the development of a strengths‐based model to promote cognitive health over the life‐course. This research will facilitate early recognition of people at risk, improve timely diagnosis and care for Indigenous peoples, their families, and communities.

## AUTHOR CONTRIBUTIONS

Huong X. T. Nguyen, Dina LoGiudice, Bridgette J. McNamara, and Rosie Watson planned this systematic review. Huong X. T. Nguyen and Catherine Voutier were responsible for database searches. Huong X. T. Nguyen and Kate Bradley screened search results, reviewed full texts, extracted information, and completed quality assessments for included articles. Dina LoGiudice and Bridgette J. McNamara assisted to achieve consensus. Roslyn Malay provided feedback on the cultural appropriateness of the review. Huong X. T. Nguyen drafted the manuscript that was reviewed by all co‐authors prior to submission.

## CONFLICT OF INTEREST STATEMENT

The authors report no disclosures or conflicts of interest relevant to the manuscript. Author disclosures are available in the supporting information.

## CONSENT STATEMENT

Participant consent was not necessary for this research.

## Supporting information

Supporting information

Supporting information
