# Supplementary material for: Risk, protective, and biomarkers of dementia in Indigenous peoples: A systematic review
Source: Alzheimers Dement. 2023 Sep 25;20(1):563–92. doi: 10.1002/alz.13458 (PMC10917055; doi:10.1002/alz.13458)
Supplement: Supplementary file 1 — Supporting information [file ALZ-20-563-s001.docx]

**APPENDICES**

**Appendix A: Search algorithm**

Database: Ovid MEDLINE(R) ALL <1946 to March 14, 2022>

Searched: 15.03.22

--------------------------------------------------------------------------------

1 ((Australia* or Northern Territor* or Tasmania* or New South Wales or Victoria* or Queensland* or New Zealand or Africa* or Ethiopia* or Nigeria* or Keny* or Scandinavia* or Norw* or Denmark or Danish or Greenland* or Sapmi or Israel* or Palestin* or Pakistan* or South America* or Americas or Brazil* or Chile* or Colombia* or Panama* or Peru or Pervian* or Amazon* or Argentin* or Bolivia* or Costa Rica* or Ecuador* or French Guiana* or Guatemala* or Guyana* or Mexic* or Nicaragua* or Paragua* or Surinam* or Venezuela* or North America* or Canad* or Hawaii* or Papua New Guinea* or PNG or Asia* or China or Chinese or Myanmar* or Nepal* or Indonesia* or Papua* or Japan* or Lao* or Malaysia* or Philippine* or Taiwan* or Thailand or Thai or Russia*) adj3 (indigenous or aborigin* or native* or Indian* or nation or nations or tribe* or tribal* or band or bands or herder* or hunter* or pastoralist* or forest* or highlander* or lowlander*)).ti. (13693)

2 ((India or population) adj3 (indigenous or aborigin* or native* or nation or nations* or tribe* or tribal* or band or bands or herder* or hunter* or pastoralist* or forest* or highlander* or lowlander*)).ti. (1797)

3 ((autochthonous or first or indigenous or native or traditional) adj3 (people* or person* or nation or nations or communit* or population*)).ti,ab. (40579)

4 African Continental Ancestry Group/ or exp American Native Continental Ancestry Group/ or exp Oceanic Ancestry Group/ or Indigenous Peoples/ or exp Africa/eh or exp Caribbean Region/eh or exp Central America/eh or exp Latin America/eh or South America/eh or Antarctic Regions/eh or Arctic Regions/eh or exp Asia/eh or exp Europe/eh or exp Oceania/eh (118083)

5 (Amazigh* or Kabyl* or Chenoua or Mzab or Berber or Berbers or San or Bushmen or Bushman or Basarwa or Balala or Nama or Fulani or Tuareg or Peul or Fulbe or Twa or Pygmy or Pygmies or Mbororo or Kirdi or Aka or Litho or Mbuti or Afar or Kunama or Saho or Nara or Majang or Majengir or Anuak or Gumuz or Bodi or Mursi or Suri or Fang or Mpongwe or Mbede or Punu or Bareke or Bateke or Bakota or Obamba or Baka or Babongo or Bakoya or Baghame or Barimba or Akoula or Akwoa or Bakouyi or Bavarama or Ogiek or Sengwer or Yiaku or Waata or Awer or Boni or Turkana or Rendille or Borana or Maasai or Samburu or Ilchamus or Somali or Gabra or Pokot or Endorois or Toubou or Songhay or Peuls or Ovatue or Ovatjimba or Ovahimba or Damara or Nama or Khwe or Hai**om or hoan* or Kung or Xun or Xoo or Kao**Aesi or Naro or Xoon or N*oha or Ovatw* or Bakola or Tswa or Tolebe or Gorgabe or Djelgobee or Bororo or Banta or Bantu or Batw* or Babongo or Baaka or Mbendjele or Mikaya or Bagombe or Babis or Khoe-San or Khoisan or Khoekhoe or Khoikhoi or Khomani or San-Khomani or Koranna or Griqua or Cushite or Nilo-Hamite or Akie or Hadzabe or Barabaig or Datoga or Benet or Ik or Karamojong or Basongora or Tshwa or Tjwa or Tsoa or Tshwao or Cua or Doma or Vadema or Tembomvura or Adivasi or Adivasis or Gowari or Chakma or Marma or Tripura or Tanchangya or Mros or Mro or Mru or Moorang or Bunong or Kuy or Santal or Khasi or Jaintia or Garos or Manipuri or Keot or Kaibarta or Pangal or Pangan or Kouy or Naxi or Tartar* or Tibetan or Uyghur* or Kazakh* or Yi or Oroqin or Gond or Bru or Reang or Mizos or Koya or Munda or Naga or Nagas or Boro or Karbi or Zo or Kuki or Dayak Meratus or Nduga or Tabi or Batak or Ainu or Okinawan* or Mon-Khmer or Sino-Tibetan or Hmong or Orang or Negrito or Semang or Senoi or Dayak or Orang Ulu or Iban or Bidayuh or Kenyah or Kayan or Kedayan or Lunbawang or Punan or Bisayah or Kelabit or Berawan).ti. (23166)

6 (Masyarakat or Kinipan or Besipae-Pubabu or Rakyat Penunggu or Osing or Sembalun or Kejaman or Ukit or Sekapan or Melanau or Penan or Anak Negeri or Dusun or Murut or Paitan or Bajau or Kachin or Karen or Karenni or Chin or Mon or Burman* or Arakan or Shan or Janajati or Newa* or Majhi* or Baram or Magar or Kiratis or Santhals or Limbu or Tharu or Raute or Kusunda or Chepang or Rajbansi or Chamar or Musahar or Vadi or Raji or Sherpa or Thakali or Igorot or Lumad or Mangyan or Dumagat or Aeta or Ifugao or Kankanaey or Pingpu or Truku or Kavalan or Siraya or Tao or Pazeh or Chao Ley or Mani or Chao-Khao or Lisu or Mien or Akha or Lahu or Lua or Thin or Khamu or Pilaga or Campo Maripe or Wichi or Qom or Quechua or Aymara or Diaguita or Chiquitano or Guarani or Moxeno or Guacamayo or Jurunna or Ra or Rikbaktsa or Mapuche* or Mapu or Iyjwaja or Chorote or Komiek or Toma or Niwackle or Chulupi or Tapy* or Tapiete or Atacameno or Licananti or Jurumi or Passe or Yuri or Wayuu or Zenu or Nasa or Pastos or Raizal or Huetar or Maleku or Bribri or Cabecar or Brunka or Ngabe or Broran or Chorotega or Andean or Kichwa or Shuar or Cofan or Shiwiar or Siekopai or Sioa or Sapara or Epera or Manta or Andes or Achuar or Waorani or Kali* or Tileuyu or Pahikweneh or Lokono or Wayampi or Wayana or Teko or Apalai or Maya* or Garifuna or Xinca or Achi or Akatec* or Awakatec* or Chalchitec* or Ch orti or Chuj or Itza or Ixil or Jacaltec* or Kaqchikel or K iche or Mam or Mopan or Poqomam or Poqomchi or Q anjob al or Q eqchi or Sakapultec* or Sipakapense or Tektitek* or Tz utujil or Uspantek* or Amerindian* or Warao or Arawak or Carib or Karinya or Wapichan or Arekuna or Makushi or Wai Wai or Patamona or Akawaio or Mazahuas or Mixtecas or Pimas or Tepehuas or Pames or Otomies or Nahuas or Amuzgas or Popolocas or Tojolabales or Zapotecas or Triquis or Yaquis or Coras or Xpujil or Nahua* or Chorotega or Cacaopera or Matagalpa or Ocanxiu or Sutiaba or Nahoa).ti. (53089)

7 (Miskitu or Sumu or Rama or Gunadule or Dule or Embera or Wounaan or Ngabe or Bugle or Naso Tjer Di or Bri Bri or Kuna or Guna or Guarani or Ache or Mbya or Pai Tavytera or Nandeva or Maskoy or Enlhet Norte or Enxet Sur or Sanapana or Angaite or Guana or Mataco or Iyjwja or Mataguayo or Nivacle or Maka or Manjui or Zamuco or Ayoreo or Yvytoso or Tomaraho or Guaicuru or Qom or Ashaninka* or Amazonia* or Kukama or Urarina or Rapa Nui or Rapanui or Kalina or Carib or Lokono or Arawak* or Tirio or Tareno or Wayana or Akoerio or Warao or Apalai or Wai-Wai or Okomoyana or Mawayana or Katuena or Tunayana or Pireuyana or Sikiiyana or Alamayana or Maraso or Awayakule or Sirewu or Upuruy or Sarayana or Kasjoeyana or Murumuruyo or Kukuyana or Piyanakoto or Saketa or Yanomami or Sanema or Yabarana or Ye kwana or Sanoma or Uwottuja or Arawak or Pemon or E Nepa or Kumanagoto or Bari or Mapoyo or Yukpa or Inuit or Kalaallit or Tunumiit or Inughuiit or Avanersuarmiut or Sami or Saami or Bedouin* or al-Kaabneh or al-Azazmeh or al-Ramadin or al-Rshaida or Jahalin or Izhma Komi or Izvatas or Tuvan* or Krymchaks or Karaim or Yakut* or Krasnoyarsk or Taimyr or Metis or Nenet* or Wet suwet en or Atikamekw or Mi kmaw or Unistoten or Tla-o-qui-aht or Nuu-chah-nuith or Metepenagiag or Gitxsan or Squamish or Tsleil-waututh or Coldwater or Stolo or Lac La Ronge or Alaska Native* or Chippewa or Ojibwe or Gwich*in or Apache or Lakota or Cheyenne or Yu*pik or Shaktoolik or Shishmaref or Kivalina or Golovin or Napakiak or Inupiat or Sioux or Zuni or Zia or Acoma or Navajo or Hopi or Picuris or Ute or Yakama or Shoshone-Bannock or Makah or Muscogee or Cherokee or Choctaw or Seminole or Chickasaw or Wampanoag or Maori or Torres Strait Islander* or Puutu Kunti Kurrama or Pinikua or Arrente or Ngaliwurru or Nungali or Watjarri or Noongar or Wongi or Anangu or Worimi or Barkindji or Wailwan or Wiradjuri or Polynesian* or Kanaka Maoli or Papua New Guinean* or Papuan* or Samonan*).ti. (13431)

8 (American adj Indian*).ti. (3780)

9 or/1-8 (239645)

10 exp Dementia/ (187923)

11 dement*.tw,kf. (133236)

12 alzheimer*.tw,kf. (169935)

13 (Primary Progressive aphasia* or Frontotemporal lobar degenerat* or Pick disease or Picks disease or Primary Progressive Nonfluenaphasia* or Korsakoff syndrome or Binswanger or guam dement* or lewy* bod* or Parkinson* disease dementia or PDD or Wernicke).ti. (8179)

14 (aMCI or MCI).tw,kf. (21882)

15 (mild cognitive or cognitive impair*).tw,kf. (79965)

16 or/10-15 (345422)

17 9 and 16 (2083)

18 Neuroimaging/ (17292)

19 (neuroimaging or imaging biomarker*).tw,kf. (63136)

20 ((brain or functional) adj3 imaging).tw,kf. (81581)

21 Positron-emission Tomography/ (58912)

22 Tomography, Emission-Computed, Single-Photon/ (32049)

23 Tomography, X-Ray Computed/ (406302)

24 Magnetic Resonance Imaging/ (447583)

25 ((positron or computer or single photon) adj3 tomograph*).tw,kf. (95879)

26 magnetic resonance imaging.tw,kf. (269058)

27 Fluorodeoxyglucose F18/ (33652)

28 (FDG or PET or SPECT or CT or MRI or fMRI).tw,kf. (756021)

29 or/18-28 (1305559)

30 Biomarkers/ (324330)

31 Amyloid.sh. (16844)

32 amyloid.tw,kf. (91631)

33 (fluid biomarker or biofluid).tw,kf. (1744)

34 cerebrospinal fluid.tw,kf. (96158)

35 (CSF or APP).tw,kf. (140402)

36 Brain/pa (107324)

37 tau Proteins/ (16842)

38 Tauopathies/ (2176)

39 tau protein*.tw,kf. (7698)

40 phospho-tau.tw,kf. (805)

41 ((intermediate adj2 filament*) or neurofilament* or tonofilament*).tw. (24345)

42 Presenilin 2/ (1082)

43 (presenilin or PSEN1 or PSEN2).tw,kf. (5234)

44 Apolipoproteins.sh. (8650)

45 (apolipoprotein or APOE).tw,kf. (57322)

46 hypometabolism.tw,kf. (3495)

47 hypoperfusion.tw,kf. (11995)

48 Temporal Lobe/ (26023)

49 temporal lobe.tw,kf. (30692)

50 or/30-49 (815654)

51 Protective Factors/ (5883)

52 ((protective or resilien*) adj3 factor*).tw,kf. (27990)

53 Risk Factors/ (914365)

54 risk factor*.tw,kf. (690188)

55 ((vascular or cerebrovascular) adj3 risk).tw,kf. (18005)

56 ((psychological or social or trauma* or environmental) adj5 risk*).tw,kf. (56126)

57 Adverse Childhood Experiences/ (2242)

58 Age Factors/ (471368)

59 Sex Factors/ (277593)

60 exp Socioeconomic Factors/ (486227)

61 "Social Determinants of Health"/ (5373)

62 (social determinant* or socioeconomic*).tw,kf. (128394)

63 (illiterac* or low literacy).tw,kf. (3851)

64 Sedentary Behavior/ (12270)

65 sedentary.tw,kf. (36301)

66 Cardiovascular Diseases/ (164858)

67 ((cardiovascular or heart) adj3 disease*).tw,kf. (401931)

68 Diabetes Mellitus/ (128331)

69 diabetes.tw,kf. (601932)

70 Epilepsy/ (80707)

71 epilep*.tw,kf. (154115)

72 Hypertension/ (247727)

73 (hypertens* or high blood pressure).tw,kf. (487888)

74 Obesity/ (200142)

75 (obesity or obese).tw,kf. (348304)

76 Smoking/ (145998)

77 (smoking or smoker*).tw,kf. (279280)

78 or/51-77 (3870460)

79 29 or 50 or 78 (5566627)

80 17 and 79 (969)

81 (animals not (humans and animals)).sh. (4937686)

82 80 not 81 (912)

***************************

**Appendix B: NIH Quality Assessment Tool for Observational Cohort and Cross-Sectional studies**

| Criteria | Yes | No | Other (CD, NR, NA)* |
| --- | --- | --- | --- |
| 1. Was the research question or objective in this paper clearly stated? |  |  |  |
| 2. Was the study population clearly specified and defined? |  |  |  |
| 3. Was the participation rate of eligible persons at least 50%? |  |  |  |
| 4. Were all the subjects selected or recruited from the same or similar populations (including the same time period)? Were inclusion and exclusion criteria for being in the study prespecified and applied uniformly to all participants? |  |  |  |
| 5. Was a sample size justification, power description, or variance and effect estimates provided? |  |  |  |
| 6. For the analyses in this paper, were the exposure(s) of interest measured prior to the outcome(s) being measured? |  |  |  |
| 7. Was the timeframe sufficient so that one could reasonably expect to see an association between exposure and outcome if it existed? |  |  |  |
| 8. For exposures that can vary in amount or level, did the study examine different levels of the exposure as related to the outcome (e.g., categories of exposure, or exposure measured as continuous variable)? |  |  |  |
| 9. Were the exposure measures (independent variables) clearly defined, valid, reliable, and implemented consistently across all study participants? |  |  |  |
| 10. Was the exposure(s) assessed more than once over time? |  |  |  |
| 11. Were the outcome measures (dependent variables) clearly defined, valid, reliable, and implemented consistently across all study participants? |  |  |  |
| 12. Were the outcome assessors blinded to the exposure status of participants? |  |  |  |
| 13. Was loss to follow-up after baseline 20% or less? |  |  |  |
| 14. Were key potential confounding variables measured and adjusted statistically for their impact on the relationship between exposure(s) and outcome(s)? |  |  |  |
| Quality Rating (Good, Fair, or Poor) |  |  |  |

*CD, cannot determine; NA, not applicable; NR, not reported

**Appendix C: Quality Assessment Results**

| Question | ***Borenstein et al., 2007 [16]*** | ***Carty et al. 2020*** | ***Chen et al., 2020 [17]*** | ***Derrig et al., 2020 [18]*** | ***Galasko et al., 2007 [19]*** | ***Gatz et al., 2022 [20]*** | ***Henderson et al., 2002 [21]*** | ***Huang et al., 2016 [22]*** | ***Lavrencic et al., 2022 [23]*** | ***LoGiudice et al., 2016 [24]*** | ***MacDonald et al., 2015 [14]*** | ***Marca Ysabel 2021 [25]*** | ***Ma’u et al., 2021 [15]*** | ***Pu’un et al., 2014 [26]*** | ***Radford et al., 2017 [27]*** | ***Radford et al., 2019 [28]*** | ***Rosenberg et al., 1996 [29]*** | ***Russell et al., 2022 [30]*** | ***Suchy-dicey et al., 2022*** | ***Smith et al., 2010 [31]*** | ***Smith M et al., 2020 [32]*** | ***Sundar et al., 2007 [33]*** | ***Thompson et al., 2022*** | ***Weiner et al., 2008 [35]*** | ***Weiner et al. 2009 [36]*** | ***Weiner et al., 2011 [37]*** |
| --- | --- | --- | --- | --- | --- | --- | --- | --- | --- | --- | --- | --- | --- | --- | --- | --- | --- | --- | --- | --- | --- | --- | --- | --- | --- | --- |
| 1. Was the research question or objective in this paper clearly stated? | Y | Y | Y | Y | Y | Y | Y | Y | Y | Y | Y | Y | Y | Y | Y | Y | Y | Y | Y | Y | Y | N | Y | Y | Y | Y |
| 1. The study population included Indigenous peoples and was clearly specified and defined? | Y | Y | Y | Y | Y | Y | Y | Y | Y | Y | Y | Y | Y | Y | Y | Y | Y | Y | Y | Y | Y | Y | Y | Y | Y | Y |
| 1. All subjects selected or recruited from the same or similar populations (including the same time period)? Were inclusion and exclusion criteria for being in the study prespecified and applied uniformly to all participants? | Y | Y | Y | Y | Y | Y | CD | Y | Y | Y | N/A | Y | N/A | Y | Y | Y | Y | Y | Y | Y | Y | Y | N/A | Y | Y | Y |
| 1. Was a sample size justification provided? | Y | N | N | Y | Y | Y | N | Y | Y | Y | N/A | Y | N/A | Y | Y | Y | Y | Y | Y | Y | N | N | N/A | Y | Y | Y |
| 1. For the analyses in this paper, were the exposure(s) (risk/protective factors) of interest measured prior to the outcome(s) being measured (cognitive impairment/dementia) ? | N | N | N | N | N | N | N | N | Y | Y | N/A | N | N/A | N | N | N | N | N | Y | N | N | N | N/A | N | N | N |
| 1. Were the outcome measures (dependent variables) clearly defined, valid, reliable, and implemented consistently across all study participants? | Y | Y | Y | Y | Y | Y | CD | Y | Y | Y | CD | Y | CD | Y | Y | Y | Y | Y | Y | Y | N | Y | CD | Y | Y | Y |
| 1. Were key potential confounding variables measured and adjusted statistically for their impact on the relationship between exposure(s) and outcome(s) | Y | Y | Y | Y | Y | Y | N | N | Y | Y | N/A | Y | N/A | Y | Y | Y | Y | Y | Y | Y | Y | Y | N/A | Y | Y | Y |
| 1. Ethics approval was sought for this study | Y | Y | Y | Y | Y | Y | N | Y | Y | Y | CD | Y | Y | Y | Y | Y | N | Y | Y | Y | CD | Y | Y | Y | CD | Y |
| 1. There was engagement, governance or co-authorship with Indigenous communities/people/organizations | N | N | N | Y | Y | Y | Y | Y | Y | Y | N | N | Y | N | Y | Y | N | Y | Y | Y | N | N | Y | Y | Y | Y |
| 1. Were positive outcomes and strength-based factors modelled? | N | N | N | N | N | Y | N | Y | Y | N | N | N | N | N | Y | Y | N | Y | N | N | N | N | Y | Y | N | N |
| 1. Does the data support the author’s interpretation of the results? | Y | Y | Y | Y | Y | Y | Y | Y | Y | Y | Y | Y | Y | Y | Y | Y | Y | Y | Y | Y | Y | Y | Y | Y | Y | Y |
| **Quality Rating (Good/Fair/Poor)** | GOOD | FAIR | FAIR | GOOD | GOOD | GOOD | POOR | GOOD | GOOD | GOOD | GOOD | FAIR | GOOD | FAIR | GOOD | GOOD | POOR | GOOD | GOOD | GOOD | FAIR | FAIR | GOOD | FAIR | FAIR | FAIR |

N= No; Y = Yes; CD=Cannot determine; N/A=Not applicable
